# Metabolomics Study of Serum from a Chronic Alcohol-Fed Rat Model Following Administration of Defatted *Tenebrio molitor* Larva Fermentation Extract

**DOI:** 10.3390/metabo10110436

**Published:** 2020-10-29

**Authors:** Ra-Yeong Choi, Moongi Ji, Mi-Kyung Lee, Man-Jeong Paik

**Affiliations:** 1 Department of Food and Nutrition, Sunchon National University, Suncheon 57922, Korea; fkdud1304@naver.com; 2 College of Pharmacy, Sunchon National University, Suncheon 57922, Korea; wlansrl@naver.com

**Keywords:** edible insect, metabolomics, metabolic profiling analysis, gas chromatography-tandem mass spectrometry

## Abstract

We have previously showed that defatted mealworm fermentation extract (MWF) attenuates alcoholic liver injury by regulating lipid, inflammatory, and antioxidant metabolism in chronic alcohol-fed rats. The current metabolomics study was performed to monitor biochemical events following the administration of MWF (daily for eight weeks) to a rat model of alcoholic liver injury by gas chromatography-tandem mass spectrometry (GC-MS/MS). The levels of 15 amino acids (AAs), 17 organic acids (OAs), and 19 free fatty acids (FFAs) were measured in serum. Analysis of variance (ANOVA), principal component analysis (PCA), and partial least squares discriminant analysis (PLS-DA) were used to compare the levels of 51 metabolites in serum. In particular, 3-hydroxybutyric acid (3-HB), pyroglutamic acid (PG), octadecanoic acid, and docosahexaenoic acid (DHA) were evaluated as high variable importance point (VIP) scores and PCA loading scores as determined by PLS-DA and PCA, and these were significantly higher in the MWF and silymarin groups than in the EtOH group. MWF showed a protective effect from alcohol-induced liver damage by elevating hepatic β-oxidation activity, and serum 3-HB levels were significantly higher in the MWF group than in the EtOH control group. Glycine levels were higher in the MWF group than in the EtOH group, and PG levels (related to glutathione production) were also elevated, indicating a reduction in alcohol-related oxidative stress. In addition, MWF is protected from alcohol-induced inflammation and steatosis by increasing serum DHA, palmitic, and octadecanoic acid levels as compared with the EtOH group. These results suggest that MWF might attenuate alcoholic liver disease, due to its anti-inflammatory and antioxidant effects by up-regulating hepatic β-oxidation activity and down-regulating liver FFA uptake.

## 1. Introduction

Edible insects offer a promising food source for future generations, due to their rich nutrient contents, sustainability, and low environmental impacts [1]. The yellow mealworm (larva of the *Tenebrio molitor*) is one of the insects produced on an industrial scale as a food or feedstuff [2]. Many studies have demonstrated that yellow mealworms have multiple beneficial physiological effects, such as anti-obesity [3], anti-osteoporotic [4], antioxidant [5], and anti-hypertensive effects [6]. Recently, Cho et al. [7] reported that yellow mealworm alcalase hydrolysates protected AML12 mouse liver cells against reactive oxygen species. Our previous study showed for the first time that defatted mealworm fermentation extract (MWF) might attenuate alcohol-induced liver injury by regulating the lipogenic and inflammatory pathways and the antioxidant defense system and by partially altering gut microbial composition [8].

Alcoholic liver disease (ALD) is a major cause of morbidity and mortality worldwide among individuals that consistently drink large amounts of alcohol [9]. Chronic alcohol intake causes mitochondrial dysfunction, oxidative stress, defective protein metabolism, and alcoholic fatty liver, which can progress to steatohepatitis, cirrhosis, and hepatocellular carcinoma [10]. Malnutrition of proteins exacerbates abnormal amino acid metabolism, which is known to occur in alcohol-consuming patients, with reduced circulating branched amino acids (BCAAs) [11,12]. In a recent study, it was reported that not only BCAAs supply improves liver disease, but also essential amino acid supply that reduces liver damage in rats chronically administered ethanol [13]. Free fatty acids (FFAs) can damage biological membranes, and their accumulation in the liver is partly responsible for the functional and morphological changes characteristic of alcoholic liver disease [14]. Therefore, it is important to monitor amino acids (AAs), organic acids (OAs), and FFAs metabolism in ALD.

Metabolomics provides a means of characterizing metabolic phenotypes and is used to identify metabolic disorders and discover biomarkers that can be used to diagnose or monitor diseases [15]. Ma et al. [16] recently reported that 35 metabolites were significantly altered in the liver of alcohol diet compared to the isocaloric control diet mice. Dong et al. [17] also demonstrated that chronic alcohol consumption induced significant alteration of serum FAs and bile acids by metabolic pathway analysis. The previous metabolomics study reported threonine, guanidinosuccinate, and glutamine as biomarkers in plasma of humans with alcohol-induced liver injury [18]. In addition, Lian et al., also reported oleamide and myristamide as biomarkers in the serum of patients with alcoholic cirrhosis [19]. However, no systematic metabolic study has been performed in ALD. Star pattern recognition analysis is a useful tool for distinguishing metabolic abnormalities [20,21], and principal component analysis (PCA), and partial least squares-difference analysis (PLS-DA), and multivariate analysis are useful for interpreting metabolomic data sets [22]. Therefore, in this study, target metabolome studies on serum AAs, OAs, and FFAs were performed by profiling, star pattern recognition, and multivariate analyses to assess hepatoprotective effects of MWF against the development of alcoholic steatosis in the ALD rat model. We have integrated the results of this metabolic profiling and previous molecular mechanism analyses [8], providing an improved understanding for the protective effects of MWF on chronic alcohol-induced liver injury.

## 2. Results

### 2.1. Metabolic Profiling Analysis and Univariate Analysis

**Figure 1:**
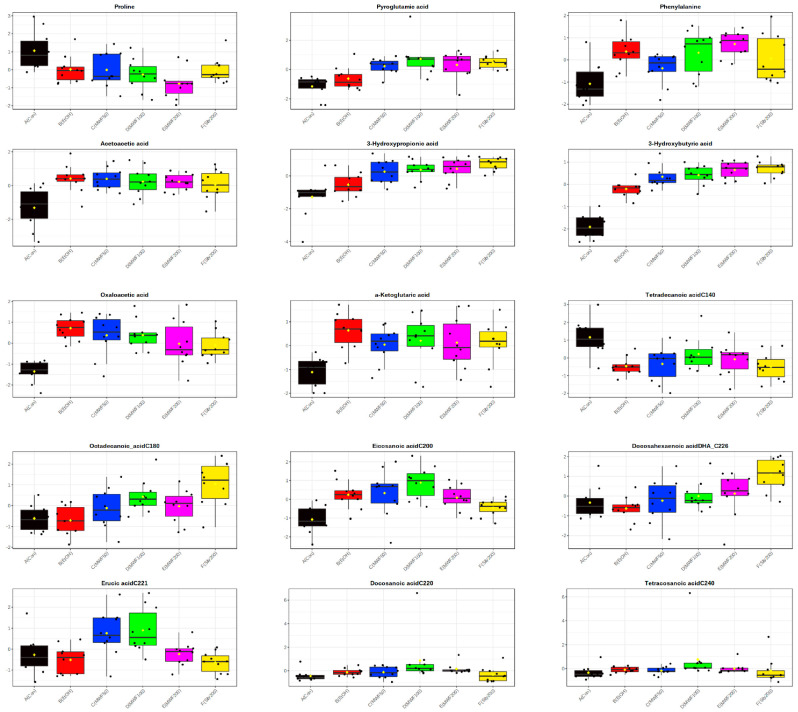
Box plots of 15 significant metabolites in ANOVA.

In serum, 51 metabolites, including 15 AAs, 17 OAs, and 19 FFAs, were determined by GC–MS/MS analysis. In all six study groups, threonine was the most abundant AA at the end of the 8-week study period, and in all groups except the MWF100 group, followed by serine and isoleucine, whereas in the MWF100 group, serine was followed by threonine. In the Con and EtOH groups, lactic acid was the most abundant OA and was followed by pyruvic acid and 3-hydroxybutyric acid (3-HB). Whereas, in the MWF and silymarin groups, lactic acid was most abundant, followed by 3-HB and pyruvic acid. In metabolic profiling analysis, acetoacetic acid (2.15~2.66-fold), 3-HB (6.48~18.53-fold), and oxalic acid (1.82~2.62-fold) showed the greatest change compared to the control group. In all groups, eicosadienoic acid (C_20:2_) was the most abundant FFA and was followed by arachidonic acid (C_20:4_), and docosatetraenoic acid (C_22:4_). Of the 51 metabolites, 3-HB (*p* < 0.001), 3-hydroxypropionic acid (*p* < 0.001), pyroglutamic acid (PG; *p* < 0.001), oxaloacetic acid (*p* < 0.001), eicosadienoic acid (*p* < 0.001), erucic acid (*p* < 0.005), octadecanoic acid (*p* < 0.006), phenylalanine (*p* < 0.007), docosanoic acid (*p* < 0.012), docosahexanoic acid (DHA; *p* < 0.012), tetradecanoic acid (*p* < 0.013), α-ketoglutaric acid (*p* < 0.019), proline (*p* < 0.019), acetoacetic acid (*p* < 0.023), and tetracosanoic acid (*p* < 0.023) were significantly different in six groups by ANOVA (Table 1, Figure 1, and Supplementary Table S1).

### 2.2. Star Pattern Recognition Analysis

**Figure 2:**
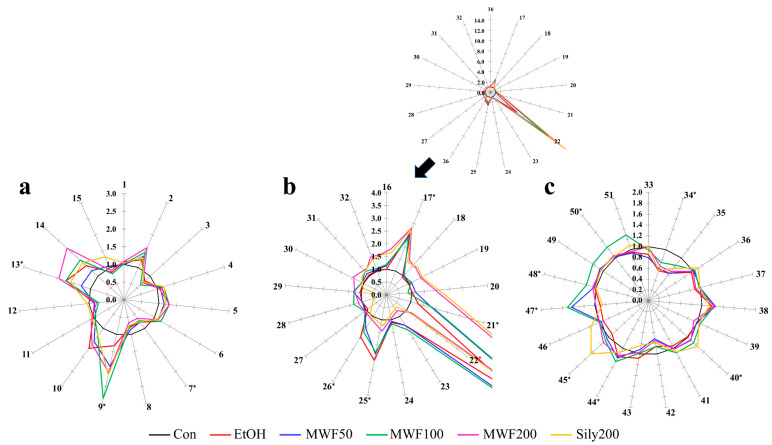
Star symbol plots of (**a**) amino acids, (**b**) organic acids, (**c**) fatty acids in serum for the Con, EtOH, MWF 50, 100, 200, and Sily200 groups mean values. Plots are drawn based on the mean levels of 15 amino acids, 17 organic acids, and 19 fatty acids as the variables after normalization to the corresponding normal mean values. * Significant metabolites in ANOVA. Ray: 1 = Alanine; 2 = Glycine; 3 = α-Aminobutyric acid; 4 = Valine; 5 = Leucine; 6 = Isoleucine; 7 = Proline; 8 = Pipecolic acid; 9 = Pyroglutamic acid; 10 = Methionine; 11 = Serine; 12 = Threonine; 13 = Phenylalanine; 14 = Aspartic acid; 15 = 4-Hydroxyproline; 16 = Pyruvic acid; 17 = Acetoacetic acid; 18 = Lactic acid; 19 = Glycolic acid; 20 = 2-Hydroxybutyric acid; 21 = 3-Hydroxypropionic acid; 22 = 3-Hydroxybutyric acid; 23 = Succinic acid; 24 = Fumaric acid; 25 = Oxaloacetic acid; 26 = α-Ketoglutaric acid; 27 = 4-Hydroxyphenylacetic acid; 28 = Malic acid; 29 = 2-Hydroxyglutaric acid; 30 = *cis*-Aconitic acid; 31 = Citric acid; 32 = Isocitric acid; 33 = Dodecanoic acid (C_12:0_); 34 = Tetradecanoic acid (C_14:0_); 35 = Palmitoleic acid (C_16:1_); 36 = Palmitic acid (C_16:0_); 37 = γ-Linolenic acid (γ-C_18:3_); 38 = Linoleic acid (C_18:2_); 39 = Oleic acid (C_18:1_); 40 = Octadecanoic acid (C_18:0_); 41 = Arachidonic acid (C_20:4_); 42 = 11-Eicosenic acid (C_20:1_); 43 = Eicosadienoic acid (C_20:2_); 44 = Eicosanoic acid (C_20:0_); 45 = Docosahexaenoic acid (DHA, C_22:6_); 46 = Docosatetraenoic acid (C_22:4_); 47 = Erucic acid (C_22:1_); 48 = Docosanoic acid (C_22:0_); 49 = Nervonic acid (C_24:1_); 50 = Tetracosanoic acid (C_24:0_) 51 = Hexacosanoic acid (C_26:0_).

Serum AA levels in the six groups at the end of the 8-week study period ranged from 0.65 to 2.85. Phenylalanine and PG levels were significantly higher in the EtOH group than in the Con group. Serum PG was significantly elevated by MWF at all doses and by silymarin, while phenylalanine was higher in the MWF100 and MWF 200 groups than in the EtOH group. Proline levels were significantly lower in the EtOH group than in the Con group and decreased dose-dependently in the MWF groups. Alteration of OA levels in all groups varied from 0.83 to 18.53. Serum acetoacetic acid, 3-hydroxypropionic acid, 3-HB, oxaloacetic acid, and α-ketoglutaric acid were significantly higher in the EtOH group than in the Con group. Of these OAs, acetoacetic, and oxaloacetic acid levels decreased dose-dependently in the MWF groups. However, 3-hydroxypropionic acid and 3-HB levels were increased dose-dependently by MWF groups. Alteration of FFA levels in all groups varied from 0.59 to 1.29. Eicosanoic acid, docosanoic acid, and tetracosanoic acid levels were significantly higher in the EtOH group than in the Con group, whereas tetradecanoic acid levels were significantly lower in the EtOH group than in the Con group and were increased by MWF at all three doses. Octadecanoic acid and DHA were significantly lower in the EtOH group than in the Con group, and their levels were elevated by MWF (at all doses) and by silymarin groups (Figure 2).

### 2.3. Multivariate Statistical Analysis

**Figure 3:**
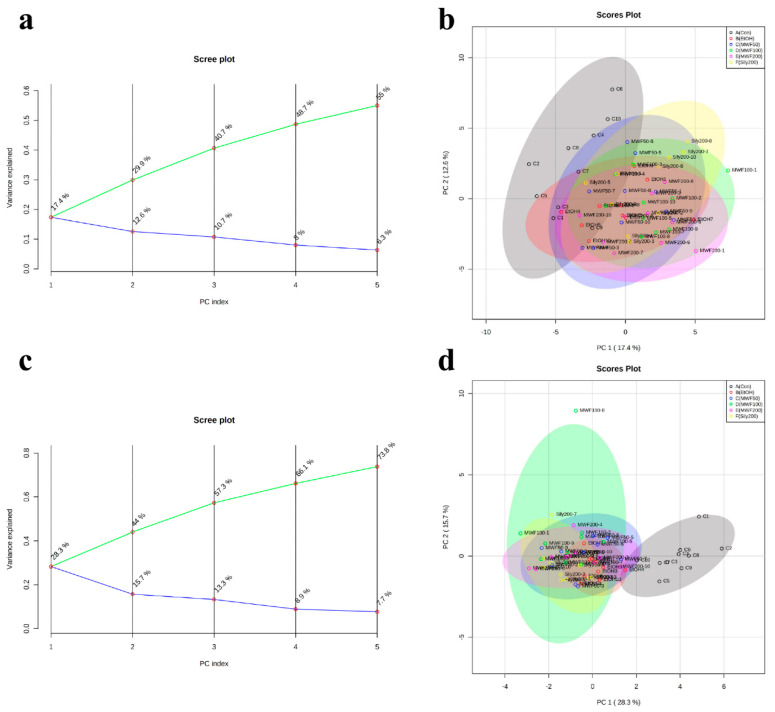
Principal component analysis (PCA) analysis for the Con, EtOH, MWF 50, 100, 200, and Sily200 groups. (**a**) Scree plot, PCA variance explained; (**b**) PCA score plot; (**c**) scree plot, and (**d**) PCA score plot of significant metabolites in ANOVA.

**Figure 4:**
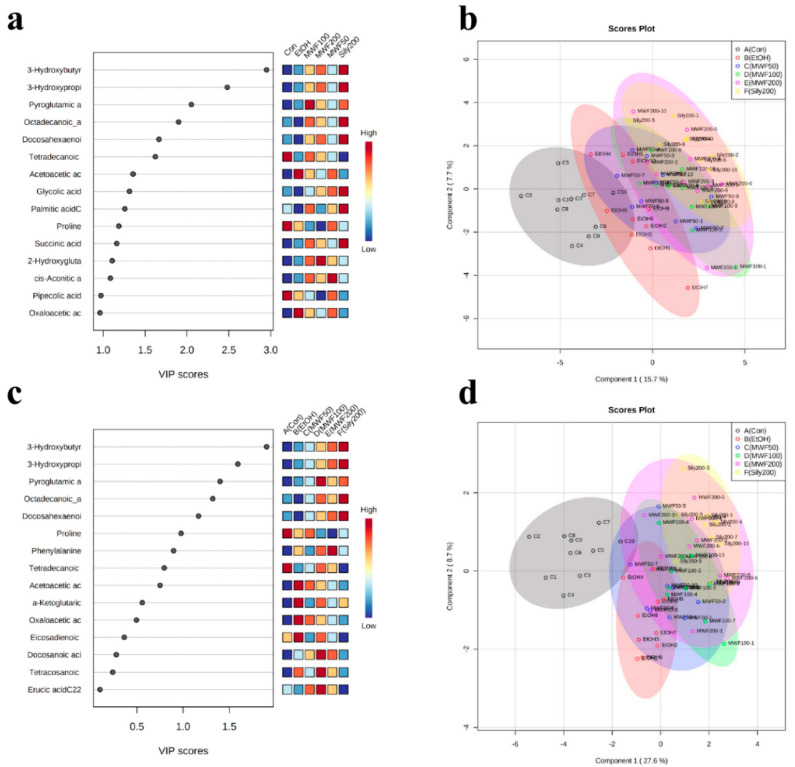
Partial least squares discriminant analysis (PLS-DA) analysis for the Con, EtOH, MWF 50, 100, 200, and Sily200 groups. (**a**) PLS-DA score plot; (**b**) variable importance analysis of top 15 metabolites; (**c**) PLS-DA score plot of significant metabolites in ANOVA; (**d**) variable importance analysis of top 15 metabolites, metabolites that are significant changes in ANOVA.

PCA (unsupervised learning) was performed using PC1 and PC2. The PCA score plot showed unclear separation between the six groups, and the two principle components (PC1 = 17.1%, PC2 = 12.4%) were associated with 29.5% of total variation (Figure 3a). However, the Con group and the EtOH group were slightly separated, whereas the Sily200 group and the MWF groups were not separated (Figure 3b). For supervised learning, PLS-DA was performed to identify biomarker candidates. The PLS-DA score plot showed unclear separation between groups with a correlation coefficient (R2) of 0.82, an accuracy of 0.45, and a cross-validation correlation coefficient (Q2) of 0.39. However, the EtOH group and the Sily200 group were slightly separated, and the MWF groups clustered with the Sily200 group (Figure 4b). PCA loading score and variable importance point (VIP) scores of PLS-DA were used to differentiate groups. Of the 51 metabolites, the five top-ranked, that is, leucine (−0.247), PG (−0.237), succinic acid (−0.235), isoleucine (−0.231), and octadecanoic acid (−0.230) were evaluated in the PCA loading score of PC1. Octadecanoic acid (2.524), 3-HB (2.391), 3-hydroxypropionic acid (2.384), PG (2.375), and DHA (1.974) were evaluated with high VIP scores by PLS-DA (Figure 4a and Table 2).

## 3. Discussion

We previously demonstrated that MWF ameliorated ALD by reducing gene expression associated with triglyceride and cholesterol synthesis and protein expression associated with the nuclear factor-κB pathway, as well as increasing hepatic glutathione (GSH) content in chronic alcohol-fed rats [8]. The MWF has more free amino acid contents than non-fermentation (data not shown). The most abundant amino acids of MWF were glutamic acid, leucine, and alanine, which were known to have therapeutic effects against liver disease [13,23,24,25,26]. Therefore, in this study, we monitored metabolic changes related to the hepatoprotective effect of MWF by performed profiling analyses on serum OAs, AAs, and FFAs.

We found that the metabolite most changed by alcohol consumption was 3-HB, which was higher in the EtOH group than in the Con group. 3-HB is a ketone body, and ketone bodies are produced predominantly in the hepatic mitochondrial matrix from β-oxidation-derived acetyl-CoA and serve as energy sources in extrahepatic tissues [27]. A previous study reported that 3-HB (3 mmol/kg, intraperitoneal injection) could protect against acute alcoholic hepatitis by enhancing the expression of the anti-inflammatory IL−10 gene and enhancing the M2 phenotype of hepatic macrophages [28]. In this study, the 3-HB level of MWF (50, 100, and 200 mg/kg) and silymarin (200 mg/kg) dose-independently was elevated by compared with the EtOH group by 2.1-, 2.1-, 2.7-, 2.9-fold, respectively. Ajmo et al. [29] reported that resveratrol increased 3-HB levels, which were increased by alcohol. The authors considered this increase might induce fatty acid oxidation and lead to ketone body production. Li et al. [30] showed that EtOH administration with dietary nicotinic acid supplementation increased serum 3-HB levels compared with the alcohol supplementation group, indicating that an elevated hepatic NAD^+^ level led to mitochondrial fatty acid β-oxidation. Our results showed that hepatic β-oxidation activity in the MWF groups (MWF50: 3.185 ± 0.484; MWF100: 4.517 ± 0.529; MWF200: 5.737 ± 0.788 nmol/min/mg protein) were dose-dependently increased as compared with the EtOH group (3.061 ± 0.455 nmol/min/mg protein; Supplementary Figure S1). Moreover, the Sily200 group (5.474 ± 0.533 nmol/min/mg protein) had activity similar to the MWF200 group. These results indicate increased 3-HB production by MWF may reflect the greater β-oxidation activity.

In this study, eight OAs related to tricarboxylic acid (TCA) cycle were up-regulated in alcohol-fed rats, and in particular, oxaloacetic acid and α-ketoglutaric acid levels increased significantly. Citric acid, *cis*-aconitic acid, isocitric acid, succinic acid, fumaric acid, and malic acid levels also showed increasing tendencies in the MWF100 and MWF200 groups as compared with the EtOH group, probably due to increased TCA cycle activity. However, the oxaloacetic acid level was dose-dependently reduced, and the α-ketoglutaric acid level was dose-independently decreased by the MWF, which were similar to those of the silymarin. Interestingly, serum liver damage markers, AST and ALT activities showed a positive correlation with oxaloacetic acid (AST; r = 0.618, *p* < 0.01, ALT; r = 0.478, *p* < 0.01) and α-ketoglutaric acid (AST; r = 0.586, *p* < 0.01, ALT; r = 0.508, *p* < 0.01). The previous study reported that plasma α-ketoglutaric acid could act as a predictor in morbidly obese patients with fatty liver disease [31]. These results indicated that the decrease of oxaloacetic acid and α-ketoglutaric acid in serum by MWF might be associated with the decrease of AST and ALT.

A recent study suggests that potential pathways associated with alcoholic liver injury, including the D-glutamine, D-glutamate, cysteine, and methionine metabolisms in the liver [16]; but we did not find the same metabolisms in the serum, which may be because the serum or tissues, animal species, alcohol dose, and duration were different. In the present study, chronic alcohol consumption was elevated PG levels compared to the Con group about 1.3-fold, however, MWF (50, 100, and 200 mg/kg) or silymarin (200 mg/kg) supplementation were further increased PG levels by 1.5-, 2.2-, 1.6-, 1.6-fold, respectively, compared with the EtOH group. PG is an intermediate metabolite of the γ-glutamyl cycle and is converted into glutamate, which resynthesizes GSH using ATP-dependent enzymes, including glutamate cysteine ligase and GSH synthase [32]. Interestingly, the PG of human placental extracts has been reported to promote liver regeneration by inducing DNA synthesis in rat primary hepatocytes via the mitogen-activated protein kinase (MAPK) pathway [33]. Since PG is more stable than glutamine or glutamic acid, it is used in care products that increase GSH production [34]. Metadoxine, which consists of PG and vitamin B6, appears to be effective at treating acute alcohol intoxication and for improving liver function following chronic alcoholism [35]. Serum glycine contents were higher in all three MWF and silymarin groups than in the EtOH group. Glycine is the final precursor amino acid required for GSH synthesis and binds to γ-glutamylcysteine to form GSH [36]. We previously found that alcohol consumption significantly reduced hepatic GSH contents and that MWF supplementation to alcohol-administered rats effectively increased hepatic GSH contents to more than that observed in the Con group [8]. GSH is a non-enzymatic antioxidant and a redox regulator in cells [36]. It is a tripeptide composed of glutamate, cysteine, and glycine [37], and plays a key role in the detoxifications of reactive oxygen species, reactive nitrogen species, and xenobiotics in cells [38]. Although we did not observe a relation between serum PG and hepatic GSH levels in the present study, our results suggest that MWF or silymarin supplementation up-regulate GSH synthesis by increasing the level of PG, a precursor of glutamate. Another previous study reported that valine, leucine, and phenylalanine levels were significantly increased in the serum following chronic alcohol intake, while they were slightly increased in our result [39].

Serum FFA metabolic profiles revealed that MWF (dose-dependent manner) and silymarin significantly increased serum DHA levels that were decreased by alcohol. Interestingly, both n−3 and n−6 polyunsaturated fatty acids (PUFAs) are components of cell membranes and precursors of biologically active substances [40], and PUFAs deficiency is commonly found in patients with ALD [41]. A previous study reported that dietary DHA attenuated alcohol-induced hepatosteatosis by down-regulating lipogenesis and inflammatory cytokine levels [42]. N−3 PUFAs can inhibit inflammatory mediators, such as protein kinases (c-jun N-terminal kinases, MAPK, p38), nuclear factor κB, and cytokines (tumor necrosis factor-α, interleukin (IL)−1β, IL−6, etc.), and reduce lipid biosynthesis by down-regulating sterol regulatory element binding protein 1c in several metabolic diseases [43]. Wang et al. [40] demonstrated that n−3 PUFAs, including DHA and eicosapentaenoic acid (EPA), alleviated ALD by reducing FFA uptake from adipose tissue by the liver. In the present study, long-chain saturated FFA serum levels, including palmitic acid and octadecanoic acid, were lower in the EtOH group than in the Con group, and MWF (at all doses) and silymarin increased these levels. In our previous study, hepatic FFA uptake-related gene expressions (fatty acid transport protein 5 (FATP5) and a cluster of differentiation [36]) were greater in the EtOH group than in the Con group, while MWF tended to reduce these expressions and silymarin significantly reduced FATP5 expression [8]. We speculate that MWF and silymarin inhibited FFAs influx into the liver, and that this increased serum FFA levels. Consequently, increased DHA levels in the MWF and silymarin groups appeared to protect alcohol-fed rats from inflammation and steatosis.

The current study was performed based on a targeted metabolomics approach of 51 metabolites rather than full metabolite analysis in ALD. In this metabolomics results, metabolic change of MWF showed a similar pattern to the metabolism of silymarin with a hepatoprotective effect in a rat model with alcoholic liver injury. In the previous study, we reported for the hepatoprotective effect of MWF [8]. Thus, the present metabolomics results may explain for the efficacy of MWF in the liver after alcohol intake. In further study, comprehensive metabolomics analysis for various metabolites with large samples in liver tissues is necessary for biomarker detection and understanding of altered metabolism related to the efficacy of MWF.

## 4. Materials and Methods

### 4.1. Chemicals and Reagents

AA standards, OA standards, FFA standards, internal standards (IS; norvaline, ^13^C_1_-leucine, ^13^C_1_-phenylalanine, ^13^C_2_-succinic acid, 3,4-dimethoxybenzoic acid, and lauric-d_2_-acid), ethyl chloroformate (ECF), and triethylamine (TEA) were purchased from Sigma-Aldrich (St. Louis, MO, USA). *N*-Methyl-*N*-*tert*-butyldimethylsilyl trifluoroacetamide (MTBSTFA) was obtained from Pierce (Rockford, IL, USA). Diethyl ether, ethyl acetate, toluene, and dichloromethane were purchased from Kanto Chemical Co. Inc. (Chuoku, Tokyo, Japan), and other reagents, including sulfuric acid, sodium hydroxide, and sodium chloride, were manufactured by Deajung (Gyeongido, Korea). All chemicals were analytical reagent grade.

### 4.2. Preparation of Serum from ALD Rat Model

Serum for GC-MS/MS analysis was acquired from ALD rats, as previously described [8]. Sprague-Dawley rats (4-week-old, males) were obtained from Orient Bio Inc. (Seoul, Korea). Rats were housed individually in stainless-steel cages in a controlled room (20 ± 2 °C and 50 ± 5% humidity) under a 12 h light-dark cycle. Animals were fed a chow diet and water *ad libitum*. After two weeks of acclimatization, they were divided into six groups of ten rats, as follows; (1) Con, an isocaloric normal liquid diet, (2) EtOH, an alcohol liquid diet, (3) MWF50, the alcohol liquid diet plus 50 mg MWF/kg BW/day, (4) MWF100, the alcohol liquid diet plus 100 mg MWF/kg BW/day, (5) MWF200, the alcohol liquid diet plus 200 mg MWF/kg BW/day, (6) Sily200 (positive control), the alcohol liquid diet plus 200 mg of silymarin (Sigma-Aldrich, Co., St. Louis, MO, USA)/kg BW/day. The silymarin has been used as a hepatoprotective agent to treat liver disease in Asia, Southern Europe, and America [44]. Liquid diets were based on the Lieber-DeCarli formulation and provided 1 kcal/mL. MWF or silymarin was dissolved in distilled water and given orally once a day, while the Con and EtOH groups were administered with distilled water. The experimental period lasted for eight weeks. Blood samples were collected from the inferior vena cava at the end of the experimental period. Serum was obtained by centrifugation (3000 rpm, 15 min, 4 °C) and stored at −80 °C until analysis. Animal care and the protocols used were approved by The Sunchon National University Institutional Animal Care and Use Committee (SCNU_IACUC−2018−12).

### 4.3. Gas Chromatography-Tandem Mass Spectrometry (GC-MS/MS)

GC-MS/MS analyses of AAs, OAs, and FFAs were performed using a GCMS-TQ8040 interfaced with a triple quadrupole mass spectrometer (Shimadzu Corp., Kyoto, Japan) in electron impact mode at 70 eV. The column used was an Ultra−2 (25 m × 0.20 mm I.D., 0.11 μm film thickness) capillary column (Agilent Technologies, Santa Clara, CA, USA). Ion source, injector, and interface temperatures were 230, 260, and 300 °C, respectively. Helium was used as the carrier gas at a constant flow rate of 0.5 mL/min. Samples (1 μL) were injected in split-injection mode (10:1). For AA analysis, the oven temperature was programmed as follows; 140 °C for 3 min, 140 °C to 300 °C at a rate of 8 °C min, and 300 °C for 5 min. For OA and FFA analyses, the following program was used; 100 °C for 2 min, 100 °C to 250 °C at10 °C/min, 250 °C to 300 °C at 20 °C/min, and 300 °C for 5 min.

### 4.4. Sample Preparation for Serum AA Profiling Analysis

Profiling analysis of AAs was performed by GC-MS using ethoxylcarbonyl (EOC)-tert-butyldimethylsilyl (TBDMS) derivatives, as previously described [21,45]. Briefly, proteins were removed using acetonitrile to 50 μL of serum containing norvaline, ^13^C_1_-leucine, and ^13^C_1_-phenylalanine as ISs (0.2, 0.4, and 0.5 μg, respectively). After centrifugation, the supernatant was spiked into deionized water (1 mL), dichloromethane (2.0 mL) containing ECF (20 μL) was added, and the pH was adjusted to ≥ 12 with 5.0 M sodium hydroxide. The two-phase EOC reaction was performed with vortexing for 10 min. The pH was then adjusted to ≤ 2 with 10% H_2_SO_4_, and the mixture was saturated with sodium chloride and sequentially extracted with diethyl ether (3 mL) and ethyl acetate (2 mL). Extracts were evaporated to dryness under a gentle stream of nitrogen (40 °C). Prior to GC-MS/MS analysis, TBDMS derivatives were produced in toluene (15 μL), MTBSTFA (20 μL), and TEA (5 μL) mixture for 1 h at 60 °C. Derivatives were transferred to an auto vial and analyzed directly by GC–MS/MS in multiple reaction monitoring (MRM) mode.

### 4.5. Sample Preparation for Serum OA Profiling Analysis

Profiling analysis of OAs was performed using methoxime (MO)-TBDMS derivatives by GC-MS, as previously described [21,46]. Briefly, proteins were removed using acetonitrile to 50 μL of serum containing ^13^C_2_-succinic acid, 3,4-dimethoxybenzoic acid as ISs (0.5 and 0.1 μg). After centrifugation, the supernatants were spiked into deionized water (1 mL), methoxyamine hydrochloride (1 mg) was then added, and pH was adjusted to ≥ 12 with 5.0 M sodium hydroxide. MO derivatives for carbonyl groups of OAs were produced by reacting at 60 °C for 1 h. After the MO reaction, pH was adjusted to pH ≤ 2 with 10% H_2_SO_4_, and the mixture saturated with sodium chloride and sequentially extracted using diethyl ether (3 mL) and ethyl acetate (2 mL). TEA (5 μL) was then added to extracts, which were then evaporated to dryness under a gentle stream of nitrogen (40 °C). Prior to GC-MS/MS analysis, TBDMS derivatives were produced in a toluene (10 μL) and MTBSTFA (20 μL) mixture for 60 min at 60 °C. Derivatives were transferred to auto vials and analyzed directly by GC–MS/MS in MRM mode.

### 4.6. Sample Preparation for Serum FFA Profiling Analysis

Profiling analysis of FFAs was performed by GC-MS of TBDMS derivatives, as previously described [21,47]. Briefly, proteins were removed using acetonitrile to 50 μL of serum containing lauric-d_2_-acid as IS (0.1 μg). After centrifugation, the supernatant was spiked into deionized water (1 mL), adjusted to pH ≤ 2 with 10% H_2_SO_4_, saturated with sodium chloride, and sequentially extracted using diethyl ether (3 mL) and ethyl acetate (2 mL). TEA (5 μL) was added to extracts and evaporated to dryness under a gentle stream of nitrogen (40 °C). Prior to GC-MS/MS analysis, TBDMS derivatives were produced in a toluene (10 μL) and MTBSTFA (20 μL) mixture for 60 min at 60 °C. Derivatives were transferred to auto vials and analyzed directly by GC–MS/MS in MRM mode.

### 4.7. Star Pattern Recognition Analysis and Statistical Analysis

Levels of the 51 metabolites in rat serum were determined using calibration curves. The amount mean levels in the five experimental groups were normalized versus the Con group. Star graph was drawn using Microsoft Excel (2010) using normalized mean values [20,21]. ANOVA was used to determine the significances of intergroup differences in metabolite levels. ANOVA was conducted using IBM SPSS Statistics 20 (IBM Corporation, Armonk, NY). Multivariate statistical analysis was performed by PCA (unsupervised learning) and PLS-DA (supervised learning). PCA was used to detect data trends and pattern analyses. PLS-DA was used to search for biomarker candidates that differentiated the Con, EtOH, MWF, and silymarin groups. Multivariate analyses were performed using log 10-transformed, mean-centered, and auto-scaled data using Metaboanalyst (https://www.metaboanalyst.ca). The validity of the PLS-DA model was verified using correlation coefficients (R_2_) and cross-validation correlation coefficients (Q_2_).

## 5. Conclusions

**Figure 5:**
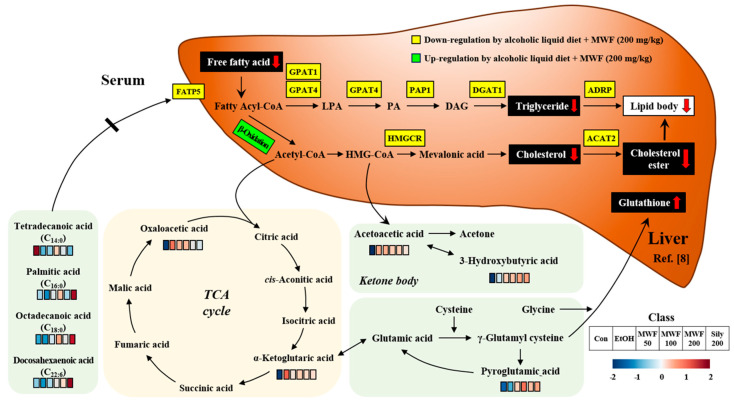
Predicted association indicating key findings of MWF from serum metabolic profiling and hepatic molecular mechanism in chronic alcohol-fed rats [8]. The red bars indicate an increased level of metabolite compared to the Con group. The blue bars indicate a reduced level of metabolite compared to the Con group. The white bars indicate no significant differences of metabolite between the treated-group and the Con group. Levels of altered metabolites showed in Table 1 and Supplementary Table S1.

Various statistical approaches were used to identify metabolites that differentiated the six study groups. 3-HB, PG, octadecanoic acid, and DHA were found to have high VIP, and PCA loading scores by PLS-DA and PCA, and their levels were significantly higher in the MWF and silymarin groups than in the EtOH group. We suggest the protective effects of MWF on alcohol-induced liver injury are associated with these 3-HB, PG, octadecanoic acid, and DHA increases and may be mediated by regulations of β-oxidation activity, inflammation, GSH production, and liver FFA uptake (Figure 5). We believe the present metabolomic study will be useful for monitoring the effectiveness of MWF treatment in alcohol-fed rats.

## Figures and Tables

**Table 1:** Levels of 15 significant metabolites in serum from Con, EtOH, MWF50, 100, 200, and Sily200 groups. MWF, mealworm fermentation extract.

No.	Metabolite	Concentration (μg/Serum of 50 μL)						Normalized Value ^a^					*p*-Value ^b^	
		Con	EtOH	MWF 50	MWF 100	MWF 200	Sily 200	EtOH	MWF 50	MWF 100	MWF 200	Sily 200	ANOVA	FDR ^c^
7	Proline	1.65 ± 0.43	1.28 ± 0.24	1.28 ± 0.28	1.20 ± 0.24	1.07 ± 0.22	1.27 ± 0.23	0.78	0.78	0.73	0.65	0.77	0.005	0.019
9	Pyroglutamic acid	0.47 ± 0.13	0.62 ± 0.28	0.90 ± 0.21	1.33 ± 1.14	0.98 ± 0.34	1.01 ± 0.22	1.32	1.92	2.85	2.09	2.16	<0.001	0.001
13	Phenylalanine	0.67 ± 0.27	1.15 ± 0.35	0.85 ± 0.20	1.16 ± 0.41	1.30 ± 0.27	1.07 ± 0.49	1.73	1.28	1.74	1.95	1.60	0.001	0.007
17	Acetoacetic acid	0.44 ± 0.27	1.18 ± 0.61	1.11 ± 0.45	1.06 ± 0.55	0.97 ± 0.29	0.95 ± 0.46	2.66	2.52	2.40	2.20	2.15	0.007	0.023
21	3-Hydroxypropionic acid	0.30 ± 0.14	0.39 ± 0.12	0.55 ± 0.18	0.57 ± 0.14	0.59 ± 0.16	0.67 ± 0.11	1.31	1.88	1.94	2.01	2.25	<0.001	0.001
22	3-Hydroxybutyric acid	0.26 ± 0.17	1.67 ± 0.70	3.53 ± 2.62	3.58 ± 1.60	4.59 ± 1.83	4.79 ± 1.87	6.48	13.67	13.87	17.77	18.53	<0.001	<0.001
25	Oxaloacetic acid	0.02 ± 0.00	0.06 ± 0.01	0.05 ± 0.02	0.05 ± 0.02	0.04 ± 0.02	0.04 ± 0.01	2.62	2.36	2.30	2.03	1.82	<0.001	0.001
26	α-Ketoglutaric acid	0.20 ± 0.04	0.39 ± 0.10	0.31 ± 0.08	0.34 ± 0.12	0.33 ± 0.14	0.32 ± 0.10	1.94	1.55	1.71	1.67	1.60	0.005	0.019
34	Tetradecanoic acid (C_14:0_)	0.10 ± 0.03	0.06 ± 0.01	0.06 ± 0.02	0.07 ± 0.02	0.06 ± 0.02	0.06 ± 0.01	0.59	0.63	0.75	0.68	0.59	0.001	0.006
40	Octadecanoic acid (C_18:0_)	7.56 ± 0.62	7.48 ± 0.68	8.10 ± 1.00	8.68 ± 0.94	8.17 ± 0.83	9.45 ± 1.27	0.99	1.07	1.15	1.08	1.25	0.001	0.006
44	Eicosanoic acid (C_20:0_)	0.03 ± 0.00	0.03 ± 0.00	0.03 ± 0.01	0.04 ± 0.00	0.03 ± 0.00	0.03 ± 0.00	1.18	1.20	1.28	1.15	1.08	<0.001	0.001
45	Docosahexaenoic acid (DHA, C_22:6_)	2.32 ± 0.57	2.12 ± 0.34	2.41 ± 0.68	2.52 ± 0.53	2.65 ± 0.65	3.36 ± 0.70	0.91	1.04	1.09	1.14	1.45	0.002	0.012
47	Erucic acid (C_22:1_)	0.01 ± 0.01	0.01 ± 0.00	0.02 ± 0.01	0.02 ± 0.01	0.01 ± 0.00	0.01 ± 0.00	0.90	1.44	1.51	0.98	0.86	0.001	0.005
48	Docosanoic acid (C_22:0_)	0.02 ± 0.00	0.02 ± 0.00	0.02 ± 0.00	0.02 ± 0.01	0.02 ± 0.00	0.02 ± 0.00	1.04	1.04	1.19	1.07	1.01	0.002	0.012
50	Tetracosanoic acid (C_24:0_)	0.02 ± 0.00	0.02 ± 0.00	0.02 ± 0.00	0.02 ± 0.01	0.02 ± 0.00	0.02 ± 0.00	1.04	1.03	1.25	1.05	1.04	0.003	0.013

^a^ Values normalized to corresponding control mean values. ^b^ ANOVA at 95% confidence level. ^c^ False Discovery Rate.

**Table 2:** The PCA loading score and variable importance point (VIP) score of PLS-DA.

No.	Metabolite	Unsupervised Learning		Supervised Learning
		PCA Loading Score		PLS-DA
		PC1	PC2	VIP Score ^a^
1	Alanine	−0.137	0.110	0.240
2	Glycine	−0.043	0.314	0.557
3	α-Aminobutyric acid	−0.025	−0.033	0.850
4	Valine	−0.219	−0.187	0.828
5	Leucine	−0.247	0.021	0.356
6	Isoleucine	−0.231	−0.101	0.185
7	Proline	0.048	−0.203	0.734
8	Pipecolic acid	0.197	−0.036	0.705
9	Pyroglutamic acid	−0.237	−0.002	2.375
10	Methionine	−0.011	0.079	0.491
11	Serine	0.034	0.203	0.365
12	Threonine	0.001	−0.171	0.064
13	Phenylalanine	−0.112	0.265	0.323
14	Aspartic acid	−0.047	0.280	0.008
15	4-Hydroxyproline	0.002	−0.167	0.707
16	Pyruvic acid	−0.100	0.003	0.615
17	Acetoacetic acid	−0.056	0.092	0.660
18	Lactic acid	−0.178	−0.001	0.223
19	Glycolic acid	−0.107	−0.034	1.438
20	2-Hydroxybutyric acid	−0.142	−0.037	0.471
21	3-Hydroxypropionic acid	−0.183	0.020	2.384
22	3-Hydroxybutyric acid	−0.194	0.124	2.391
23	Succinic acid	−0.235	0.047	1.114
24	Fumaric acid	−0.193	0.115	0.147
25	Oxaloacetic acid	−0.205	0.115	0.334
26	α-Ketoglutaric acid	−0.216	0.058	0.233
27	4-Hydroxyphenylacetic acid	0.014	0.049	0.080
28	Malic acid	−0.174	0.160	0.572
29	2-Hydroxyglutaric acid	−0.146	0.078	0.988
30	*cis*-Aconitic acid	−0.051	0.093	1.083
31	Citric acid	−0.092	0.164	0.650
32	Isocitric acid	−0.096	0.153	0.569
33	Dodecanoic acid (C_12:0_)	−0.016	−0.064	0.463
34	Tetradecanoic acid (C_14:0_)	0.046	−0.079	0.695
35	Palmitoleic acid (C_16:1_)	0.080	−0.184	0.143
36	Palmitic acid (C_16:0_)	0.113	−0.155	1.812
37	γ-Linolenic acid (γ-C_18:3_)	−0.180	−0.249	0.935
38	Linoleic acid (C_18:2_)	−0.061	−0.135	0.514
39	Oleic acid (C_18:1_)	−0.115	−0.097	0.522
40	Octadecanoic acid (C_18:0_)	−0.230	−0.184	2.524
41	Arachidonic acid (C_20:4_)	−0.210	−0.115	1.359
42	11-Eicosenic acid (C_20:1_)	−0.081	−0.220	0.351
43	Eicosadienoic acid (C_20:2_)	−0.099	−0.240	0.859
44	Eicosanoic acid (C_20:0_)	−0.186	−0.192	0.569
45	Docosahexaenoic acid (DHA, C_22:6_)	−0.180	0.007	1.974
46	Docosatetraenoic acid (C_22:4_)	−0.145	−0.131	1.129
47	Erucic acid (C_22:1_)	−0.103	−0.163	0.835
48	Docosanoic acid (C_22:0_)	−0.093	−0.038	0.550
49	Nervonic acid (C_24:1_)	−0.083	0.042	0.620
50	Tetracosanoic acid (C_24:0_)	−0.058	0.004	0.575
51	Hexacosanoic acid(C_26:0_)	0.030	0.015	0.532

^a^ Variable importance in projection score.

## Supplementary Materials

The following are available online at https://www.mdpi.com/2218-1989/10/11/436/s1, Figure S1: Effects of MWF on the hepatic β-oxidation activity in chronic alcohol-fed rats, Table S1: Levels of metabolites in serum from con, EtOH, MWF-50, 100, 200, and sily200 groups.
