# Supplementary material for: Metabolomics Study of Serum from a Chronic Alcohol-Fed Rat Model Following Administration of Defatted Tenebrio molitor Larva Fermentation Extract
Source: Metabolites. 2020 Oct 29;10(11):436. doi: 10.3390/metabo10110436 (PMC7693418; doi:10.3390/metabo10110436)
Supplement: Supplementary file 1 [file metabolites-10-00436-s001.pdf]

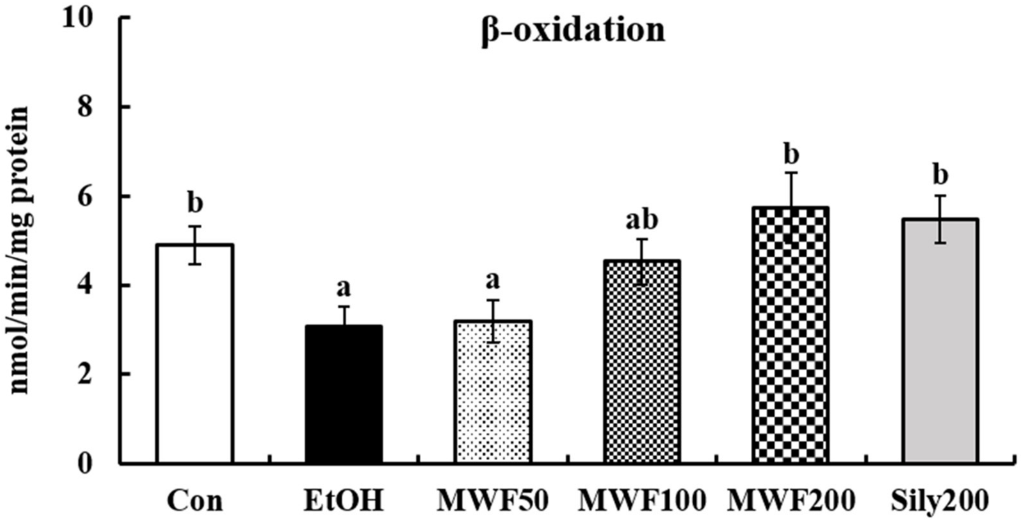

Supplementary Figure S1. Effects of MWF on the hepatic β-oxidation activity in chronic alcohol-fed rats. Values was expressed as mean±SE (n=10). Statistical significance was determined by one-way ANOVA, followed by Duncan's multiple range test. Values not sharing a common letter (a, b) above the bars are significantly different among the groups at $p < 0.05$.

Supplementary Table S1. Levels of metabolites in serum from con, EtOH, MWF-50, 100, 200, and sily200 groups

No.	Metabolite	Concentration (μg/ serum of 50 μL)						Normalized value[a]					*p*-value[b]
		Con	EtOH	MWF50	MWF100	MWF200	Sily200	EtOH	MWF50	MWF100	MWF200	Sily200	
1	Alanine	2.18 ± 0.32	2.35 ± 0.38	2.18 ± 0.39	2.23 ± 0.31	2.37 ± 0.49	2.28 ± 0.42	1.08	1.00	1.02	1.08	1.04	0.823
2	Glycine	0.63 ± 0.28	0.78 ± 0.25	0.84 ± 0.29	0.92 ± 0.41	1.01 ± 0.26	0.83 ± 0.37	1.24	1.35	1.47	1.61	1.32	0.144
3	α-Aminobutyric acid	0.20 ± 0.07	0.16 ± 0.07	0.15 ± 0.06	0.12 ± 0.07	0.14 ± 0.05	0.15 ± 0.04	0.80	0.75	0.63	0.72	0.75	0.191
4	Valine	1.70 ± 0.44	1.91 ± 0.36	1.84 ± 0.40	2.02 ± 0.36	1.78 ± 0.27	2.04 ± 0.54	1.12	1.08	1.19	1.05	1.20	0.373
5	Leucine	1.73 ± 0.31	2.22 ± 0.37	1.99 ± 0.37	2.23 ± 0.34	2.24 ± 0.34	2.07 ± 0.30	1.29	1.15	1.29	1.30	1.20	0.140
6	Isoleucine	2.67 ± 0.62	3.25 ± 0.68	2.98 ± 0.58	3.25 ± 0.57	2.99 ± 0.40	2.93 ± 0.42	1.22	1.12	1.22	1.12	1.10	0.261
7	Proline	1.65 ± 0.43	1.28 ± 0.24	1.28 ± 0.28	1.20 ± 0.24	1.07 ± 0.22	1.27 ± 0.23	0.78	0.78	0.73	0.65	0.77	0.005
8	Pipecolic acid	0.02 ± 0.01	0.02 ± 0.00	0.02 ± 0.01	0.02 ± 0.01	0.02 ± 0.00	0.02 ± 0.01	0.79	0.86	0.77	0.66	0.73	0.079
9	Pyroglutamic acid	0.47 ± 0.13	0.62 ± 0.28	0.90 ± 0.21	1.33 ± 1.14	0.98 ± 0.34	1.01 ± 0.22	1.32	1.92	2.85	2.09	2.16	<0.001
10	Methionine	0.11 ± 0.03	0.19 ± 0.08	0.16 ± 0.11	0.14 ± 0.06	0.17 ± 0.10	0.14 ± 0.06	1.69	1.45	1.27	1.55	1.26	0.300
11	Serine	3.26 ± 0.48	3.30 ± 0.40	3.04 ± 0.51	3.15 ± 0.42	3.52 ± 0.51	3.43 ± 1.28	1.01	0.93	0.96	1.08	1.05	0.628
12	Threonine	12.09 ± 3.54	10.25 ± 3.74	10.32 ± 4.94	8.79 ± 3.26	9.47 ± 3.14	12.65 ± 3.94	0.85	0.85	0.73	0.78	1.05	0.193
13	Phenylalanine	0.67 ± 0.27	1.15 ± 0.35	0.85 ± 0.20	1.16 ± 0.41	1.30 ± 0.27	1.07 ± 0.49	1.73	1.28	1.74	1.95	1.60	0.001
14	Aspartic acid	0.17 ± 0.10	0.25 ± 0.12	0.21 ± 0.09	0.29 ± 0.14	0.38 ± 0.13	0.28 ± 0.25	1.44	1.23	1.68	2.18	1.58	0.060
15	4-Hydroxyproline	0.58 ± 0.26	0.49 ± 0.14	0.60 ± 0.29	0.47 ± 0.24	0.52 ± 0.27	0.76 ± 0.32	0.85	1.04	0.81	0.91	1.33	0.145
16	Pyruvic acid	1.68 ± 0.38	1.69 ± 0.37	1.91 ± 0.44	2.00 ± 0.64	2.08 ± 0.45	1.82 ± 0.35	1.01	1.14	1.19	1.24	1.08	0.272
17	Acetoacetic acid	0.44 ± 0.27	1.18 ± 0.61	1.11 ± 0.45	1.06 ± 0.55	0.97 ± 0.29	0.95 ± 0.46	2.66	2.52	2.40	2.20	2.15	0.007
18	Lactic acid	36.14 ± 5.62	33.72 ± 12.03	35.59 ± 7.56	37.33 ± 10.45	38.05 ± 11.33	34.41 ± 6.33	0.93	0.98	1.03	1.05	0.95	0.897
19	Glycolic acid	4.72 ± 0.73	4.58 ± 0.90	5.20 ± 0.80	5.06 ± 0.77	5.11 ± 0.97	5.46 ± 0.72	0.97	1.10	1.07	1.08	1.16	0.191
20	2-Hydroxybutyric acid	0.14 ± 0.05	0.14 ± 0.09	0.16 ± 0.08	0.12 ± 0.05	0.16 ± 0.05	0.16 ± 0.05	1.02	1.15	0.85	1.16	1.21	0.524
21	3-Hydroxypropionic acid	0.30 ± 0.14	0.39 ± 0.12	0.55 ± 0.18	0.57 ± 0.14	0.59 ± 0.16	0.67 ± 0.11	1.31	1.88	1.94	2.01	2.25	<0.001
22	3-Hydroxybutyric acid	0.26 ± 0.17	1.67 ± 0.70	3.53 ± 2.62	3.58 ± 1.60	4.59 ± 1.83	4.79 ± 1.87	6.48	13.67	13.87	17.77	18.53	<0.001
23	Succinic acid	1.03 ± 0.40	1.45 ± 0.48	1.45 ± 0.31	1.67 ± 0.45	1.55 ± 0.49	1.63 ± 0.61	1.41	1.41	1.62	1.50	1.58	0.054
24	Fumaric acid	0.03 ± 0.01	0.04 ± 0.02	0.04 ± 0.02	0.04 ± 0.02	0.04 ± 0.01	0.04 ± 0.01	1.10	1.06	1.15	1.21	1.06	0.897
25	Oxaloacetic acid	0.02 ± 0.00	0.06 ± 0.01	0.05 ± 0.02	0.05 ± 0.02	0.04 ± 0.02	0.04 ± 0.01	2.62	2.36	2.30	2.03	1.82	<0.001
26	α-Ketoglutaric acid	0.20 ± 0.04	0.39 ± 0.10	0.31 ± 0.08	0.34 ± 0.12	0.33 ± 0.14	0.32 ± 0.10	1.94	1.55	1.71	1.67	1.60	0.005
27	4-Hydroxyphenylacetic acid	0.002 ± 0.001	0.002 ± 0.001	0.003 ± 0.001	0.003 ± 0.001	0.004 ± 0.004	0.002 ± 0.000	0.92	1.05	1.07	1.59	0.83	0.221
28	Malic acid	0.13 ± 0.08	0.13 ± 0.07	0.13 ± 0.07	0.17 ± 0.10	0.19 ± 0.09	0.15 ± 0.07	1.00	1.00	1.35	1.52	1.16	0.308
29	2-Hydroxyglutaric acid	0.09 ± 0.03	0.10 ± 0.03	0.12 ± 0.05	0.12 ± 0.03	0.14 ± 0.03	0.11 ± 0.02	1.05	1.31	1.28	1.49	1.19	0.063
30	*cis*-Aconitic acid	0.02 ± 0.00	0.02 ± 0.00	0.02 ± 0.00	0.02 ± 0.00	0.02 ± 0.00	0.02 ± 0.00	1.03	1.08	1.08	1.07	1.07	0.223
31	Citric acid	0.26 ± 0.09	0.27 ± 0.08	0.30 ± 0.11	0.33 ± 0.10	0.32 ± 0.09	0.30 ± 0.08	1.04	1.13	1.25	1.23	1.12	0.551
32	Isocitric acid	0.04 ± 0.01	0.04 ± 0.01	0.04 ± 0.01	0.05 ± 0.01	0.05 ± 0.01	0.04 ± 0.01	1.04	1.06	1.10	1.10	1.07	0.684
33	Dodecanoic acid ($C_{12:0}$)	0.02 ± 0.00	0.02 ± 0.00	0.02 ± 0.00	0.02 ± 0.00	0.02 ± 0.01	0.02 ± 0.00	0.86	0.80	0.94	1.00	1.00	0.130
34	Tetradecanoic acid ($C_{14:0}$)	0.10 ± 0.03	0.06 ± 0.01	0.06 ± 0.02	0.07 ± 0.02	0.06 ± 0.02	0.06 ± 0.01	0.59	0.63	0.75	0.68	0.59	0.001
35	Palmitoleic acid ($C_{16:1}$)	0.24 ± 0.09	0.15 ± 0.04	0.16 ± 0.05	0.20 ± 0.08	0.17 ± 0.04	0.18 ± 0.04	0.64	0.64	0.83	0.69	0.76	0.070
36	Palmitic acid ($C_{16:0}$)	7.73 ± 1.03	7.42 ± 0.64	7.82 ± 0.66	8.01 ± 0.47	7.67 ± 0.47	8.57 ± 1.07	0.96	1.01	1.04	0.99	1.11	0.060
37	γ-Linolenic acid (γ -$C_{18:3}$)	0.03 ± 0.00	0.02 ± 0.00	0.03 ± 0.00	0.03 ± 0.00	0.03 ± 0.00	0.03 ± 0.00	0.89	0.99	0.98	0.91	0.99	0.204
38	Linoleic acid ($C_{18:2}$)	3.50 ± 0.70	4.16 ± 0.72	4.38 ± 1.10	4.31 ± 0.59	4.34 ± 0.60	4.01 ± 0.67	1.19	1.25	1.23	1.24	1.14	0.101
39	Oleic acid ($C_{18:1}$)	6.03 ± 1.55	6.04 ± 0.65	5.93 ± 0.93	6.35 ± 0.63	5.57 ± 0.78	6.21 ± 0.97	1.00	0.98	1.05	0.92	1.03	0.582
40	Octadecanoic acid ($C_{18:0}$)	7.56 ± 0.62	7.48 ± 0.68	8.10 ± 1.00	8.68 ± 0.94	8.17 ± 0.83	9.45 ± 1.27	0.99	1.07	1.15	1.08	1.25	0.001
41	Arachidonic acid ($C_{20:4}$)	18.44 ± 3.33	17.54 ± 2.13	18.66 ± 2.45	20.20 ± 2.08	18.72 ± 2.93	19.94 ± 3.09	0.95	1.01	1.10	1.01	1.08	0.262
42	11-Eicosenic acid ($C_{20:1}$)	0.13 ± 0.05	0.11 ± 0.04	0.09 ± 0.05	0.11 ± 0.03	0.09 ± 0.04	0.10 ± 0.03	0.86	0.71	0.86	0.75	0.78	0.421
43	Eicosadienoic acid ($C_{20:2}$)	54.81 ± 19.30	59.08 ± 18.96	49.99 ± 19.91	53.83 ± 12.20	53.01 ± 14.47	45.93 ± 9.04	1.08	0.91	0.98	0.97	0.84	0.581
44	Eicosanoic acid ($C_{20:0}$)	0.03 ± 0.00	0.03 ± 0.00	0.03 ± 0.01	0.04 ± 0.00	0.03 ± 0.00	0.03 ± 0.00	1.18	1.20	1.28	1.15	1.08	<0.001
45	Docosahexaenoic acid (DHA, $C_{22:6}$)	2.32 ± 0.57	2.12 ± 0.34	2.41 ± 0.68	2.52 ± 0.53	2.65 ± 0.65	3.36 ± 0.70	0.91	1.04	1.09	1.14	1.45	0.002
46	Docosatetraenoic acid ($C_{22:4}$)	10.33 ± 2.18	9.50 ± 2.34	9.86 ± 2.33	10.15 ± 2.67	10.27 ± 1.57	12.02 ± 1.43	0.92	0.95	0.98	0.99	1.16	0.160
47	Erucic acid ($C_{22:1}$)	0.01 ± 0.01	0.01 ± 0.00	0.02 ± 0.01	0.02 ± 0.01	0.01 ± 0.00	0.01 ± 0.00	0.90	1.44	1.51	0.98	0.86	0.001
48	Docosanoic acid ($C_{22:0}$)	0.02 ± 0.00	0.02 ± 0.00	0.02 ± 0.00	0.02 ± 0.01	0.02 ± 0.00	0.02 ± 0.00	1.04	1.04	1.19	1.07	1.01	0.002
49	Nervonic acid ($C_{24:1}$)	0.02 ± 0.00	0.02 ± 0.00	0.02 ± 0.00	0.02 ± 0.01	0.02 ± 0.00	0.02 ± 0.00	1.07	1.08	1.23	1.05	1.05	0.273
50	Tetracosanoic acid ($C_{24:0}$)	0.02 ± 0.00	0.02 ± 0.00	0.02 ± 0.00	0.02 ± 0.01	0.02 ± 0.00	0.02 ± 0.00	1.04	1.03	1.25	1.05	1.04	0.003
51	Hexacosanoic acid ($C_{26:0}$)	0.02 ± 0.00	0.02 ± 0.00	0.02 ± 0.00	0.02 ± 0.02	0.02 ± 0.00	0.02 ± 0.01	0.95	0.93	1.29	0.96	1.08	0.579

[a] Values normalized to corresponding control mean values
[b] ANOVA at 95% confidence level
